# Parkinson’s disease detection based on multi-pattern analysis and multi-scale convolutional neural networks

**DOI:** 10.3389/fnins.2022.957181

**Published:** 2022-07-27

**Authors:** Lina Qiu, Jianping Li, Jiahui Pan

**Affiliations:** School of Software, South China Normal University, Guangzhou, China

**Keywords:** Parkinson’s disease, disease detection, EEG, multi-pattern analysis, multi-scale convolutional neural networks

## Abstract

Parkinson’s disease (PD) is a complex neurodegenerative disease. At present, the early diagnosis of PD is still extremely challenging, and there is still a lack of consensus on the brain characterization of PD, and a more efficient and robust PD detection method is urgently needed. In order to further explore the features of PD based on brain activity and achieve effective detection of PD patients (including OFF and ON medications), in this study, a multi-pattern analysis based on brain activation and brain functional connectivity was performed on the brain functional activity of PD patients, and a novel PD detection model based on multi-scale convolutional neural network (MCNN) was proposed. Based on the analysis of power spectral density (PSD) and phase-locked value (PLV) features of multiple frequency bands of two independent resting-state electroencephalography (EEG) datasets, we found that there were significant differences in PSD and PLV between HCs and PD patients (including OFF and ON medications), especially in the β and γ bands, which were very effective for PD detection. Moreover, the combined use of brain activation represented by PSD and functional connectivity patterns represented by PLV can effectively improve the performance of PD detection. Furthermore, our proposed MCNN model shows great potential for automatic PD detection, with cross-validation accuracy, sensitivity, specificity, and area under the receiver operating characteristic curve all above 99%. Our study may help to further understand the characteristics of PD and provide new ideas for future PD diagnosis based on spontaneous EEG activity.

## Introduction

Parkinson’s disease (PD) is the second most common neurodegenerative disease and the most prevalent movement disorder in the world (Surguchov, 2021). Its main pathological change is the progressive degeneration of neurons in the substantia nigra pars compacta, which results in a range of motor (e.g., tremor, bradykinesia, rigidity, and postural gait disturbance) and non-motor symptoms (e.g., depression and sleep disturbance) (Lotankar et al., 2017). These symptoms become more and more severe with the development of the disease, which seriously affects the patients’ daily life and work. Especially in the middle and late stages, patients basically lose their ability to take care of themselves, and their follow-up care will bring a heavy burden to the patient’s family and society. For PD patients, there is currently neither a cure nor an effective way to slow the progression of the disease. Most PD patients are usually not diagnosed until the middle and advanced stages of the disease, when the patient has missed the optimal treatment period. It is believed that early diagnosis and effective preventive treatment can delay the onset of specific symptoms and significantly improve the quality of life of patients (Ugrumov, 2020). Therefore, it is very necessary to find an effective and high-accuracy method for the early diagnosis of PD.

Currently, the diagnosis of PD mainly relies on the medical observation and clinical symptom evaluation of the patient by a specialist. E.g., physicians examine patients according to some clinical diagnostic criteria, such as the Unified Parkinson’s Disease Rating Scale (UPDRS) (Mantovani et al., 2018). However, this traditional diagnostic method is susceptible to subjectivity, leading to possible misclassification. Furthermore, symptoms of early PD can be mild and unnoticed. Therefore, the diagnostic method based on symptom observation is difficult to accurately diagnose early PD. A United Kingdom autopsy study found that the misdiagnosis rate of PD based on symptom observation was as high as 24% (Pagan, 2012). Currently, early diagnosis of PD remains challenging.

With the rapid development of neuroimaging technology, there are more and more studies using non-invasive brain functional imaging technology as an auxiliary means to detect brain diseases, such as positron emission tomography (PET), functional Magnetic Resonance Imaging (fMRI), and electroencephalography (EEG) (Arbabshirani et al., 2017). Among them, EEG technology is very popular in the field of clinical neuroscience due to its advantages of portability, low cost and high time resolution, and is widely used in the auxiliary diagnosis of neurological diseases and the research of brain function rehabilitation. In recent years, more and more studies have used EEG for PD detection, and EEG is considered as a potential diagnostic modality that can identify the unique features of PD (Wang et al., 2020). Using this modality, the researchers observed that PD patients had higher rates of EEG abnormalities than normal older adults. Compared with healthy controls (HCs), PD patients had slower resting-state changes in brain oscillatory activity (Soikkeli et al., 1991; Neufeld et al., 1994) and phase-amplitude coupling (de Hemptinne et al., 2013; Swann et al., 2015; Gong et al., 2021), as well as reduced β and γ power (Pezard et al., 2001). Moreover, PD EEG abnormalities were also manifested in changes in functional brain connectivity. Compared with HCs, PD patients have reduced connectivity in the α-β band, increased connectivity in the γ band (Conti et al., 2022), and exhibit loss of frontotemporal connectivity (Hassan et al., 2017).

In recent years, with the increasing maturity of artificial intelligence and pattern recognition technologies, tools based on computer-aided diagnosis have brought great help to the early diagnosis of PD. An increasing number of studies were devoted to coupling various advanced machine learning (ML) or deep learning (DL) algorithms and EEG signals to detect PD automatically (Mei et al., 2021). For the ML-based methods, Menorca et al. (2017) used random forests to classify the EEG signals of 50 PD patients and 41 HCs with a recognition accuracy of 78.0%. Based on high-order statistical feature extraction technology and support vector machine (SVM) classifier, Yuvaraj et al. (2018) obtained a 99.62% accuracy in distinguishing EEG signals from PD patients and HCs. Oliveira et al. (2020) used random forest and feature selection techniques to obtain over 99% classification accuracy for PD patients and HCs. Khoshnevis and Sankar (2021) proposed a PD detection method based on higher-order statistical techniques and achieved a classification accuracy of 87.00% using an ensemble RUSBoosted trees classifier in 20 PD patients and 20 HCs. Gunduz (2021) proposed an efficient dimensionality reduction method to detect PD and reported 95.70% accuracy using a SVM classifier. Barua et al. (2021) used the proposed graph-based aspirin feature extractor and k-nearest neighbor classifier to automatically detect PD, and the results obtained 93.57% accuracy in the classification of healthy and PD off medication (PD_OFF), and 95.48% in the classification of HC and PD on medication (PD_ON). Khare et al. (2021a) used the least squares SVM on five different features extracted from the tunable Q-factor wavelet transform of a resting-state EEG dataset to discriminate HC from PD patients with and without medications at an accuracy of 96% and 97.7%. Most recently, Anjum et al. (2020) developed a linear-predictive-coding EEG Algorithm to encode EEG time series into features for PD detection, and obtained reliable performance with 85.7% diagnostic accuracy, 85.2% area under curve (AUC) of receiver operating characteristics (ROC), 85.7% sensitivity, and 85.7% specificity. Lee et al. (2022) proposed a PD prediction method using the Hjorth parameter and gradient boosting decision tree algorithm, which differentiated PD patients from HCs with 89.3% accuracy and 0.912 AUC of ROC.

With the development of deep learning methods in the last few years, more and more studies have also explored EEG-based automatic PD detection (Tanveer et al., 2022). Gil-Martín et al. (2019) detected PD patients by using a convolutional neural network (CNN) to analyze subjects’ the drawing movements and achieved 96.5% accuracy. Oh et al. (2020) proposed a 13-layer CNN architecture to classify the resting-state EEG signals of 20 PD patients and 20 HCs with an accuracy of 88.25%. Khare et al. (2021b) applied a 2D-CNN to the Smoothed Pseudo-Wigner Ville Distribution transformation on two resting state EEG datasets with validation accuracies of 99.9% and 100%. Loh et al. (2021) have also applied a 2D-CNN on the Gabor transform of a resting-state EEG dataset in order to classify subjects into HC and PD_OFF and PD_ON with an accuracy of 99.5%. Shaban (2021) used an artificial neural network-based framework and three EEG spatial channels to screen subjects as PD patients and HCs with 98% accuracy, 97% sensitivity, and 100% specificity. More recently, they recently introduced a 20-layer CNN-based deep learning method to identify HC, PD_OFF, and PD_ON using the wavelet domain of resting-state EEG with 99.9% accuracy (Shaban and Amara, 2022).

Taken together, there are considerable evidences that EEG-based brain activation and functional connectivity differ significantly between PD patients and HCs, and ML and DL techniques show great potential for automated PD detection. However, there are still some issues worth further exploration in this field. First, the functional brain features identified in the current study that may be used for PD detection are diverse, lacking valid and reliable characterizations. Second, most of the previous studies only based on the feature of a single pattern (activation pattern or brain network pattern) to characterize and identify PD, which was difficult to fully reflect the brain functional characteristics of PD patients. Finally, there is still a lack of efficient and robust automatic PD detection models. Although many of the previously proposed ML and DL frameworks have shown great potential for PD detection, most have only shown good results on a specific research dataset and have not been generalized to other datasets, making it difficult to extended to clinical diagnosis of PD patients.

To further investigate the characteristics of brain activity of PD patients and achieve effective detection of PD patients, in this study, we investigated the characterization and identification of PD based on two independent resting-state EEG data. Specifically, we analyzed the differences in power spectral density (PSD) and phase-locked value (PLV) between PD patients and HCs, respectively, and applied the SVM method and the proposed model to distinguish PD (including PD_OFF and PD_ON) patients and HCs. The contributions of this study can be summarized as follows:

(1) The multi-pattern analysis of brain activation and brain functional connectivity was performed on spontaneous EEG activity in PD patients, and PD (including PD_OFF and PD_ON) was identified with high performance by combining these two complementary patterns.
(2) Two-class and three-class challenges were addressed in two independent datasets, and multiple evaluation indicators of accuracy, sensitivity, specificity, and AUC were used to provide accurate screening for PD patients.
(3) An efficient and robust deep learning-based PD detection model, multi-scale CNN (MCNN), was proposed to identify PD and HC subjects with high performance in two independent datasets, with the highest accuracy, sensitivity, specificity and AUC were higher than 99%.

## Materials and methods

### Datasets

A total of 2 public resting-state EEG datasets were used in this study. The first dataset is from OpenNeuro, obtained in the Aron lab at the University of California at San Diego, and further curated by the Swann lab at the University of Oregon (Rockhill et al., 2020). The second dataset is a public dataset (Anjum et al., 2020) recorded by the University of Iowa (UI; Iowa City, Iowa). The following is a detailed description of the two datasets.

#### UC San Diego dataset

The dataset included EEG data from 15 right-handed PD patients (8 females, mean age 62.6 ± 8.3 years) and 16 HCs (9 females, 63.5 ± 9.6 years). The patients were recruited from Scripps Clinic in La Jolla, CA, United States, all with either mild or moderate PD, and HCs were volunteers from the local community. In this dataset, each PD patient visited the laboratory for two sessions, ON and OFF medication. The order of the visits was counter-balanced between patients. For the OFF session, the time between the last dose of dopaminergic medication and the visit was 12 h or more, and for the ON session, the patients took their usual morning dose before coming for the session. Therefore, the EEG data of all 15 PD patients included data on both on and off medication (denoted in the paper as PD_ON and PD_OFF, respectively). In EEG measurements, a 32-channel Biosemi Active Two EEG system was used to collect subjects’ resting-state EEG data at a sampling frequency of 512 Hz for at least 3 mins. The positions of the 32-channel EEG electrodes are Fp1, AF3, F7, F3, FC1, FC5, T7, C3, CP1, CP5, P7, P3, Pz, PO3, O1, Oz, O2, PO4, P4, P8, CP6, CP2, C4, T8, FC6, FC2, F4, F8, AF4, Fp2, Fz, and Cz. The details of these datasets are described in Rockhill et al. (2020).

#### Iowa dataset

The dataset includes EEG recordings from 14 PD patients and 14 HCs. The author of this dataset did not clearly state whether the PD data here belongs to ON medication (PD_ON) or OFF medication (PD_OFF), so we named it directly as PD in this article. This EEG dataset was recorded from 0.1 to 100 Hz sintered Ag/AgCl electrodes at a sampling rate of 500 Hz on a 64-channel Brain Vision system (recorded for at least 2 mins per subject) with an online reference set to channel Pz as baseline. Therefore, Pz data is missing from this dataset (Iowa). The details of these datasets are described in Anjum et al. (2020).

In both datasets, HC participants and PD patients were demographically matched for age and sex, and there were no differences in education or any measure of pre-morbid intelligence. Moreover, there was no statistically significant difference in United Parkinson’s Disease Rating Scale (UPDRS III) scores between PD-OFF and PD-ON in the UC San Diego dataset (*P*-value ≈ 0.08). The information of the subjects is shown in Table 1.

**TABLE 1:** Parkinson’s disease and control participant demographics in the UC San Diego dataset and the Iowa dataset.

Condition	UC San Diego dataset		Iowa dataset	
	PD	Control	PD	Control
Number	15	16	14	14
Sex (female/male)	8f/7m	9f/7m	8f/6m	8f/6m
Age (mean years ± SD)	63.2 ± 8.2	63.5 ± 9.6	70.5 ± 8.7	70.5 ± 8.7
NAART	46.1 ± 6.3	49.1 ± 7.1	−	−
MMSE	28.9 ± 1.0	29.2 ± 1.1	−	−
MOCA	−	−	25.9 ± 2.7	27.2 ± 1.7
UPDRS	39.2 ± 9.7 (OFF)/ 32.7 ± 10.4 (ON)	−	13.4 ± 6.6	−
Year since Parkinson’s diagnosis	4.5 ± 3.5.	−	5.6 ± 3.2	−

NAART, Scores from the North American Adult Reading Test; MMSE, Mini Mental State Exam; MOCA, Montreal Cognitive Assessment; UPDRS, United Parkinson’s Disease Rating Scale (motor).

### Data processing

#### Data preprocessing

All EEG data were preprocessed using the EEGLAB toolbox (Delorme and Makeig, 2004). The EEG data of each channel were first band-pass filtered at 0.5∼50 Hz, and then independent component analysis (ICA) was used to remove noise interference including eye movement artifacts, channel noise, and heartbeat. For convenient and reliable comparison, we analyze the data of 32 channels in common in the two datasets, namely Fp1, AF3, F7, F3, FC1, FC5, T7, C3, CP1, CP5, P7, P3, Pz, PO3, O1, Oz, O2, PO4, P4, P8, CP6, CP2, C4, T8, FC6, FC2, F4, F8, AF4, Fp2, Fz, and Cz. For the Iowa dataset, since the data of the Pz channel was missing, we averaged the data of the surrounding 4 channels (i.e., P1, P2, CPz, and POz) as the data of the Pz channel.

For further analysis, we intercepted the first three and first 2 mins of EEG data for all subjects in the UC San Diego dataset and the Iowa dataset, respectively. Then, the data in the UC San Diego dataset (3 mins in length) and the Iowa dataset (2 mins in length) were divided into 180 time samples and 120 time samples with a time sample of 1 s. Therefore, after preprocessing, the EEG data for each subject in the UC San Diego dataset was collated to a size of 32 × 512 × 180 (channels × sampling points × time samples), while the EEG data for each subject in the Iowa dataset was organized into a size of 32 × 500 × 120 (channels × samples × time samples).

#### Multi-pattern analysis of spontaneous electroencephalography activity

In this study, in order to reflect brain function more comprehensively, we analyzed not only the features that can reflect the local activation of the brain, but also the features that can reflect the functional network of the brain. Specifically, we selected power spectral density (PSD) and phase-locked value (PLV), which are commonly used in EEG data processing, as representatives of the two patterns of local brain activation and brain functional connectivity, respectively.

Power spectral density can reflect the energy distribution of EEG in each frequency band in each brain region, and PLV can reflect the phase synchronization relationship of EEG signals in each brain region (Li et al., 2019). T The activation pattern represented by the PSD feature can capture the spontaneous power differences in various regions of the subject’s brain, while the connectivity pattern represented by the PLV feature can represent the information synchronization in each region of the subject’s brain. The combined analysis of these two complementary patterns can reflect the functional state of the brain more comprehensively and accurately.

Power spectral density refers to the concept of density to represent the distribution of signal power at each frequency point. In the PSD-based feature analysis of this study, we used a discrete Fourier transform on the preprocessed EEG data to calculate the power values for each channel in five frequency bands, i.e., δ (1–4 Hz), θ (4–8 Hz), α (8–12 Hz), β (13–30 Hz), and γ (30–48 Hz). PLV is commonly used to assess the spread of the distribution of phase angle differences between EEG electrode pairs over time, which can reflect long-range synchronous changes in neural activity in the brain (Lachaux et al., 1999). The connectivity is measured from this spread such that strongly clustered phase differences between two electrodes result in the PLV close to 1, which indicates strong connectivity between the signals. If PLV is 0, there is no phase dependence between the signals of the two EEG channels (electrodes). To obtain the PLV, we first filtered the preprocessed EEG data in the desired frequency band of interest by using a Hamming Plus A windowed FIR (Finite Impulse Response) filter (Widmann et al., 2015). In this study, we are interested in five frequency bands including δ, θ, α, β, and γ bands. Then, the instantaneous phase of the signal was calculated using the Hilbert transform. Finally, the PLV between two signals A and B was computed as follow (Bruna et al., 2018):


$${\mathrm{PLV}}_{A,B}=\frac{1}{N}\,\left|\sum_{n=1}^{N}e^{-i\,\left(\phi_{A}\,\left(t\right)-\phi_{B}\,\left(t\right)\right)}\right|$$

where *N* is the number of trials (here, we defined 1s of resting-state data as a trial), and ϕ (t) is the instantaneous phase angles of each EEG signal. We calculated the PLV for every second (one trial) of EEG data for each subject, resulting in a time series of PLVs calculated from all 32 electrode pairs and five frequency bands. Note that the Fisher transform was used in the calculation of the group mean PLV for the subjects tested.

### Classification

Based on the analyzed PSD and PLV features, we further classified PD patients (including PD_ON and PD_OFF) and HC using traditional ML methods and DL methods. For the ML method, we applied the traditional SVM algorithm, and for the deep learning method, we proposed a novel DL model based on multi-scale CNN (MCNN). Furthermore, to verify the effectiveness of multi-pattern analysis of activation features and functional connectivity features for PD detection, we combined PSD and PLV features together to form a PSD+PLV hybrid feature for classification. All features used for classification were normalized before being input to the classification model, including PSD+PLV hybrid features.

#### Support vector machine model

Support vector machine is the most classic and one of the most popular classification algorithms in ML in recent decades, and has been successfully applied to classification in various fields of pattern recognition, including PD detection. In this study, we used an SVM classifier with a linear kernel based on the popular LIBSVM toolbox (Chang and Lin, 2011) to classify feature data of PD patients and HCs.

In each classification experiment, all channel data for classified subjects were stitched together and labeled differently by group (e.g., PD and HC). E.g., in the two-class classifications of PD_ON and HC in UC San Diego dataset, HC data was labeled as 0 and PD_ON data was labeled as 1. When classifying based on the PSD features of the δ band, the PSD feature data size of each subject in the δ band is 180 (time samples) × 32 (channels), then the feature data size for PD_ON and HC classification is [180 × 15(PD_ON) + 180 × 16 (HC)] × 32 (channels), which is 5580 × 32. In the classification process, the 5580 × 32 feature data was divided into a training set (5022 × 32) and a test set (558 × 32) in a ratio of 9:1.

#### Multi-scale convolutional neural network model

**FIGURE 1:**
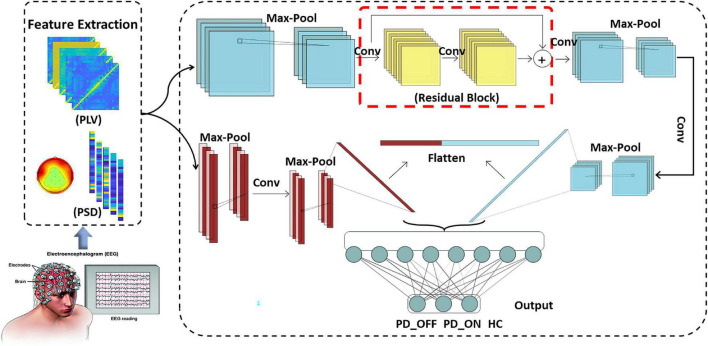
Framework diagram of multi-scale convolutional neural networks (MCNN).

To efficiently and reliably detect PD automatically, we designed a novel deep learning-based PD detection model, namely the Multi-Scale CNN (MCNN) model, as shown in Figure 1. CNN is a multi-layer perceptron that uses local connections and weight sharing to reduce the number of network training parameters. The CNN models have been successfully used in recent studies for automatic PD detection based on EEG signals (Tanveer et al., 2022). Our proposed MCNN model was improved on the traditional CNN network LeNet-5 network (Lecun et al., 1998). Compared with the LeNet-5 model, the improvement of the MCNN model is manifested in two aspects. On the one hand, the number of network layers is increased. MCNN contains a total of two network layers, which can simultaneously extract the information of the input feature matrix on one-dimensional and two-dimensional scales. On the other hand, MCNN introduces the idea of residual learning. Residual learning not only helps to extract deeper features, but also effectively solves the problem of network degradation. The detailed improvements are as follows:

(1) Multi-scale convolution:

Traditional CNN models usually perform single-scale convolution on the input features. However, accurate detection of diseases usually relies on multi-pattern and multi-scale information. Therefore, we used two different sub-networks in parallel in the MCNN model to extract the information on the one-dimensional and two-dimensional scales of the input feature matrix, respectively. The two sub-networks are parallel and have different depths, which can perform nonlinear transformations on local features with different dimensions and depths, making them more adaptable and expressive. At the end of the two sub-networks, the attribute features of different scales are flattened and spliced to obtain a feature tensor representation with higher dimension and richer local detail features.

(2) Residual learning:

Compared with traditional pattern recognition methods, deep networks have more network layers and more complex structures, so they can extract deeper features. i.e., to say, the depth of the network affects the performance of the model. However, as the depth of the network increases, the network degenerates, causing the accuracy rate to begin to saturate or even decrease. To solve this problem, we added a residual block containing a convolution kernel of size 5 × 5 (shown in the red box in Figure 1) into the first sub-network convolution process of the MCNN model to optimize the network degradation problem. The deep residual network helps to extract deeper features, and as the depth of the network increases, it can also effectively reduce the problem of vanishing gradients, resulting in more efficient use of features and enhanced feature transfer between convolutional layers.

The overall framework of MCNN is shown in Figure 1, which contains two sub-networks. We invoke residual learning after the first convolution pooling in the first sub-network. In the second sub-network, we only do convolution and pooling. Moreover, Dropout was added after each fully connected layer to prevent the model from overfitting, and finally two-class or three-class classifications were performed. The optimizer used by this model was the SGD optimizer with a learning rate of 0.001 and a decay rate of 0.1. The model was built using Keras and Pytorch backend in Python programming. The specific implementation parameters of this model are shown in Table 2.

**TABLE 2:** The implementation parameter of the multi-scale convolutional neural network (MCNN) model.

	Layer	Layer Depth	Layer Size	Activation	Parameter
Sub-Network 1	Input	−	32 × 32	−	−
	Convolution	6	5 × 5	ReLU	Padding = Same
	MaxPooling	6	2 × 2	−	−
	Convolution	16	5 × 5	ReLU	−
	Convolution	16	5 × 5	ReLU	Padding = 2
	Convolution	16	5 × 5	ReLU	Padding = 2
	MaxPooling	16	2 × 2	−	
	Convolution	120	5 × 5	ReLU	−
	MaxPooling	120	2 × 2	−	−
Sub-Network 2	Convolution	32	7 × 1	ReLU	Padding = Same
	MaxPooling	32	2 × 1	−	−
	Convolution	64	5 × 1	ReLU	−
	MaxPooling	64	2 × 1	−	−
	Dropout	−	−	−	Rate = 0.5
	Dense	84	−	−	−
	Dropout	−	−	−	Rate = 0.5
	Dense	2/3	−	−	Sigmoid/Softmax

#### Performance evaluation and statistical analysis

In the feature analysis, Student’s *t*-test is used to test the significance of differences in comparisons. In the classification experiments based on the UC San Diego dataset, we performed three sets of two-class classification using the SVM and MCNN models, respectively, for the HC group, PD_ON group, and PD_OFF group, and one set of three-class classifications using the MCNN model. In the classification based on the Iowa dataset, we used the SVM and MCNN models to classify the HC group and the PD group, respectively. Experimental results were evaluated using multiple metrics of accuracy, specificity, sensitivity, and ROC. All classification results are ten-fold cross-validation.

## Experimental results

### Results of multi-pattern analysis

In this study, we first performed PSD and PLV feature analysis in five EEG frequency bands (i.e., δ, θ, α, β, and γ bands) in PD patients and HCs in two datasets. Then the group-averaged PSD and group-averaged PLV in HC group and PD group (including PD_ON group and PD_OFF group in UC San Diego dataset), as well as the ratio of group-average and the significance of difference between groups were calculated, respectively. For UC San Diego dataset, we compared the differences in PSD features and PLV features between PD_ON, PD_OFF and HC groups, respectively. For Iowa dataset, we compared the differences in PSD features and PLV features between PD and HC groups.

**FIGURE 2:**
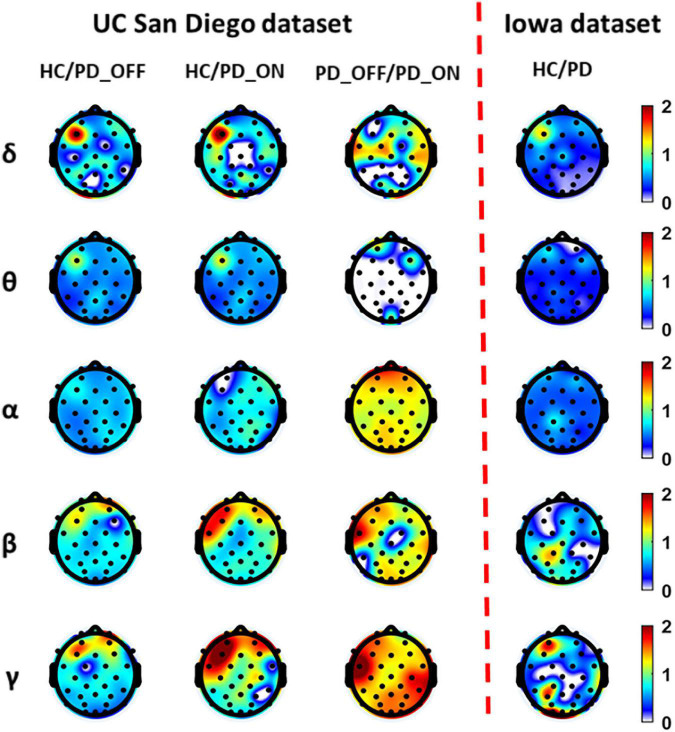
The contrast maps of group-averaged power spectral density (PSD) in five frequency bands (i.e., δ, θ, α, β, and γ bands) between the Parkinson’s disease (PD) patient group (including PD_OFF and PD_ON) and the HC group for 2 different datasets. The white areas (values less than 0 in the colorbar) indicate that the difference between the two groups is not statistically significant (i.e., *P* > 0.05). The yellow-red (values greater than 1.0 in the colorbar) area represents the ratio’s numerator with a larger PSD value than the denominator, while the cyan-blue (values greater than 0 and less than 1.0 in the colorbar) are the opposite. The darker the color, the greater the difference in PSD between the two groups.

Figure 2 presents contrast maps of group-averaged PSD in five frequency bands for HC and PD (including PD_OFF and PD_ON in UC San Diego dataset) in two datasets, showing the ratios of group-averaged PSD of the two groups and the statistically significant channel between the two groups. The first three columns in Figure 2 are the PSD contrast maps between groups in UC San Diego dataset (15 PD_OFF, 15 PD_ON, and 16 HC) for the five frequency bands (i.e., δ, θ, α, β, and γ bands), and the last column is the PSD contrast maps between groups in Iowa dataset (14 PD and 14 HC) for the five frequency bands. The white area in Figure 2 (i.e., the value less than 0 in the color bar) indicates that the difference between the two groups in this area is not statistically significant (i.e., *P* > 0.05), and other colors indicate that the difference is statistically significant (*P*-value ≤ 0.05). Among them, the yellow-red (values greater than 1.0 in the colorbar) area represents the ratio’s numerator with a larger PSD value than the denominator, while the cyan-blue (values greater than 0 and less than 1.0 in the colorbar) are the opposite. The darker the color, the greater the difference in PSD between the two groups. Take the first subplot in the first column of Figure 2 as an example, which shows the PSD difference between the HC group and the PD_OFF group in the δ band in the UC San Diego dataset. The white area in the figure represents that the PSD of the HC group and the PD_OFF group is not significantly different in this part of the area. The red area of the left prefrontal lobe represents a significant difference between the two groups and the PSD of the HC group is greater than PD_OFF, the cyan area represents a significant difference between the two groups and the PSD values of the two groups are equivalent, and the blue area represents a significant difference between the two groups and the PSD of PD_OFF is greater than that of HC. The darker the color, the greater the difference in PSD between the HC group and the PD_OFF group in the δ band.

As can be seen from Figure 2, the PSD features of PD groups (including PD_OFF and PD_ON) and HC are almost all significantly different (*P* > 0.05) in the five frequency bands of both datasets, except for the difference in θ band for PD_OFF and PD_ON in UC San Diego dataset. PD groups (including PD_OFF and PD_ON) showed stronger PSD in most channels than HC group in the θ band, while their PSD in the frontal regions in the β and γ bands was weaker than that in the HC group. In other cases, although the PSD of PD group (including PD_OFF and PD_ON) and HC group were significantly different in most channels, the activation amplitudes were comparable. For PD_OFF and PD_ON in the UC San Diego dataset, most of the channels in the α, β, and γ bands of PD_OFF show significantly stronger PSDs than PD_ON, and they are not significantly different in θ band.

**FIGURE 3:**
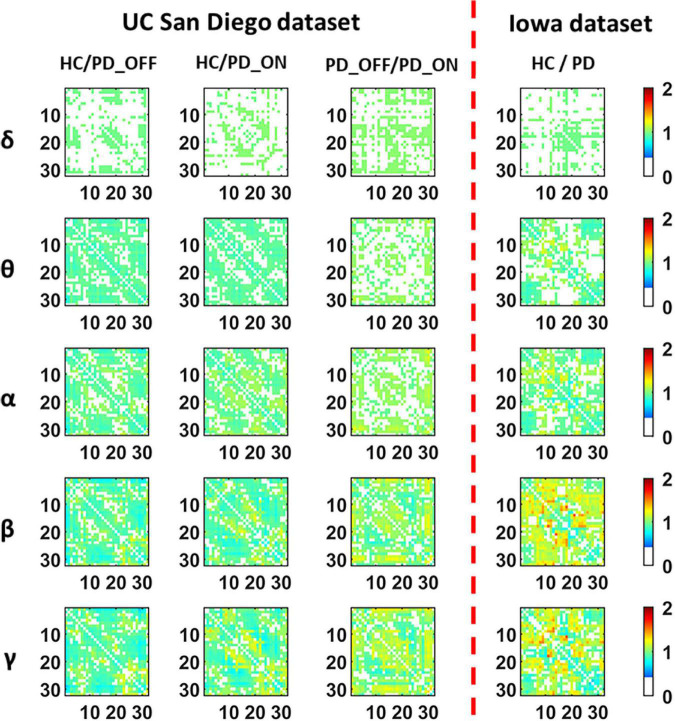
The contrast maps of group-averaged phase-locked value (PLV) in five frequency bands (i.e., δ, θ, α, β, and γ bands) between the PD patient group (including PD_OFF and PD_ON) and the HC group for 2 different datasets. The white areas (values less than 0 in the colorbar) indicate that the difference between the two groups is not statistically significant (i.e., *P* > 0.05). The yellow-red (values greater than 1.0 in the colorbar) area represents the ratio’s numerator with a larger PLV value than the denominator, while the cyan-blue (values greater than 0 and less than 1.0 in the colorbar) are the opposite. The darker the color, the greater the difference in PLV between the two groups. The numbers on the abscissa and ordinate in each subplot represent the channels of the electroencephalography (EEG).

Figure 3 presents contrast maps of group-averaged PLV in five frequency bands for HC and PD (including PD_OFF and PD_ON in UC San Diego dataset) in two datasets, showing the ratio of group-averaged PLV of the two groups and the statistically significant EEG channel pairs between the two groups. Here the group-averaged PLVs were obtained by Fisher transformation. The first three columns in Figure 3 are the PLV contrast maps between groups in UC San Diego dataset (15 PD_OFF, 15 PD_ON, and 16 HC) for the five frequency bands (i.e., δ, θ, α, β, and γ bands), and the last column is the PLV contrast maps between groups in Iowa dataset (14 PD and 14 HC) for the five frequency bands. The colorbar of Figure 3 and the meaning of the color are similar to Figure 2. From the PLV contrast maps in Figure 3, it can be seen that the HC group and the PD group (including PD_OFF and PD_ON) show significant differences (*P* ≤ 0.05) in PLV for most channel pairs in θ, α, β, and γ bands. In the Iowa dataset, PD patients exhibited slightly smaller PLVs in the β and γ bands than HCs. In the UC San Diego dataset, the difference between PD_OFF and PD_ON is also more significant in β and γ bands, and PD_OFF shows a great PLV than PD_ON.

In conclusion, in the analysis and comparison based on PSD features and PLV features, we found that PD group (including PD_OFF and PD_ON) and HC group, as well as PD_OFF and PD_ON, had significant differences in both PSD and PLV features, especially in β and γ bands are more pronounced. The PSD here can reflect the distribution of spontaneous activation of brain nerves, while the PLV can reflect the synchronization of spontaneous brain activity among brain regions. Therefore, the results of the above analysis may imply that PD and HC have significant differences in brain functional activation and functional connectivity, and this difference is especially pronounced in the β and γ bands.

### Classification results

In the second part of this study, we classified PD (including PD_OFF and PD_ON) and HC using traditional SVM algorithm based on PSD and PLV features, and used metrics such as accuracy, sensitivity, specificity, and AUC for ROC to evaluate experimental results. The following two-class classifications were first performed using SVM based on PSD and PLV features and their hybrid feature PSD+PLV, respectively: HC vs. PD_OFF, HC vs. PD_ON, PD_OFF vs. PD_ON for UC San Diego dataset; and HC vs. PD for Iowa dataset. Among them, the results of two-class classifications (accuracy, sensitivity and specificity) based on PSD and PLV features alone in five frequency bands (i.e., δ, θ, α, β, and γ bands) by using SVM are shown in Tables 3, 4, respectively. All results were obtained by ten-fold cross-validation. It can be seen that PD (including PD_OFF and PD_ON) and HC, as well as PD_OFF and PD_ON, can be effectively distinguished based on the features of PSD (Table 3) and PLV (Table 4), especially in the β and γ frequency bands. In the β and γ frequency bands, the classification accuracy based on PSD features are above 70%, and the classification accuracy based on PLV features are above 80% (the highest is 94.36% in HC vs. PD). Moreover, the classification performance based on PLV features generally outperforms PSD features.

**TABLE 3:** The results of two-class classifications based on power spectral density (PSD) features in the two datasets by using support vector machine (SVM) (unit: %, Accu. = Accuracy, Sens. = Sensitivity, Spec. = Specificity).

	HC vs. PD_OFF (UC San Diego dataset)			HC vs. PD_ON (UC San Diego dataset)			PD_OFF vs. PD_ON (UC San Diego dataset)			HC vs. PD (Iowa dataset)		
	Accu.	Sens.	Spec.	Accu.	Sens.	Spec.	Accu.	Sens.	Spec.	Accu.	Sens.	Spec.
**δ**	66.77	51.02	81.57	67.69	74.84	61.09	61.24	77.66	44.74	51.77	74.49	48.38
**θ**	73.13	60.84	84.62	74.35	64.42	83.69	58.70	75.92	41.59	76.90	64.63	89.17
**α**	73.15	57.78	87.60	71.37	55.83	85.93	62.15	84.00	40.33	71.07	54.72	87.42
**β**	78.69	72.75	84.24	82.33	82.50	82.18	72.21	77.97	66.48	75.36	65.46	83.45
**γ**	76.05	67.66	83.97	81.51	86.27	76.96	73.28	78.09	68.54	78.08	68.61	87.52

**TABLE 4:** The results of two-class classifications based on phase-locked value (PLV) features in the two datasets by using SVM (unit: %, Accu. = Accuracy, Sens. = Sensitivity, Spec. = Specificity).

	HC vs. PD_OFF (UC San Diego dataset)			HC vs. PD_ON (UC San Diego dataset)			PD_OFF vs. PD_ON (UC San Diego dataset)			HC vs. PD (Iowa dataset)		
	Accu.	Sens.	Spec.	Accu.	Sens.	Spec.	Accu.	Sens.	Spec.	Accu.	Sens.	Spec.
**δ**	55.66	54.14	57.08	56.76	52.93	60.34	54.17	51.66	56.71	62.59	68.22	56.99
**θ**	69.98	68.86	71.07	68.33	67.25	69.32	61.87	62.60	61.15	76.01	76.70	75.31
**α**	76.24	74.50	77.88	75.91	75.22	76.61	66.06	65.74	66.45	84.17	84.38	84.04
**β**	83.44	82.68	84.11	85.66	85.52	85.80	80.6	77.97	79.14	92.16	92.29	92.04
**γ**	88.12	87.11	89.07	90.30	89.88	90.66	82.63	82.82	82.47	94.36	94.10	94.63

**FIGURE 4:**
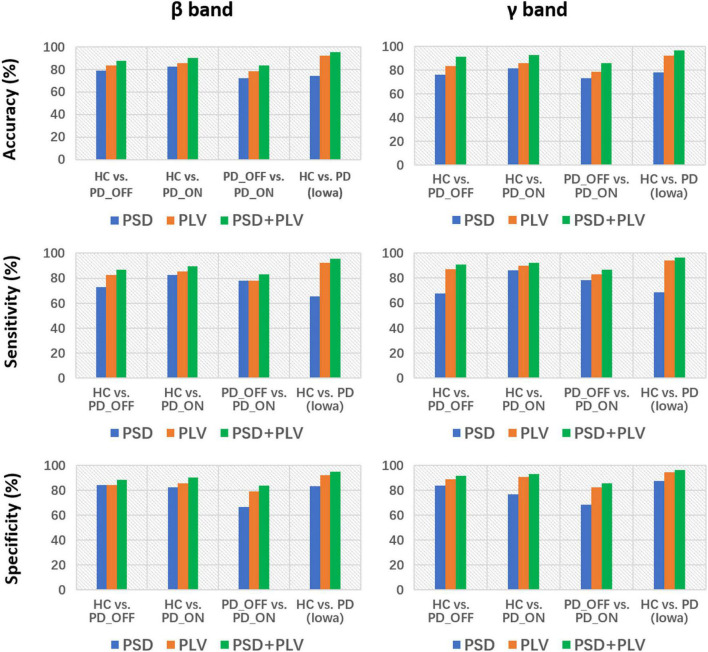
Accuracy, sensitivity, and specificity for the classification based on PSD, PLV, and PSD+PLV features in the β and γ bands by using support vector machine (SVM) for the four comparison groups (i.e., HC vs. PD_OFF, HC vs. PD_ON, PD_OFF vs. PD_ON in UC San Diego dataset, and HC vs. PD in Iowa dataset).

Moreover, we also integrated the PSD features and PLV features of each frequency band separately to form new feature matrixes PSD+PLV, and used SVM for classification, the results are shown in Table 5. Compared to using PSD and PLV features alone, combining PSD and PLV resulted in improved classification accuracy, sensitivity, and specificity, especially in the β and γ bands, as shown in Figure 4. In the cases of HC vs. PD_OFF, HC vs. PD_OFF and HC vs. PD (Iowa dataset), the accuracy, sensitivity and specificity obtained based on PSD+PLV features in the γ band were all above 90%, where HC vs. PD (Iowa dataset) had the highest classification performance with 96.43% (accuracy), 96.58% (sensitivity), and 96.28% (specificity). Figure 4 shows the comparison of the classification accuracy, sensitivity and specificity of SVM based on PSD, PLV and PSD+PLV in the β and γ bands. It can be observed from Figure 4 that in the β and γ bands, PLV can provide better classification performance than PSD, and the combination of PSD and PLV can provide better classification performance than PSD and PLV alone.

**TABLE 5:** The results of two-class classifications based on PSD+PLV features in the two datasets by using SVM (unit: %, Accu. = Accuracy, Sens. = Sensitivity, Spec. = Specificity).

	HC vs. PD_OFF (UC San Diego dataset)			HC vs. PD_ON (UC San Diego dataset)			PD_OFF vs. PD_ON (UC San Diego dataset)			HC vs. PD (Iowa dataset)		
	Accu.	Sens.	Spec.	Accu.	Sens.	Spec.	Accu.	Sens.	Spec.	Accu.	Sens.	Spec.
**δ**	64.01	60.82	67.10	66.49	65.69	67.30	59.35	62.20	56.50	54.11	67.53	41.10
**θ**	76.03	74.14	77.79	74.22	73.22	75.15	62.25	61.79	62.77	85.46	85.63	85.31
**α**	80.20	79.34	81.03	79.37	78.80	79.90	68.29	69.25	67.34	86.33	86.29	86.41
**β**	87.64	86.91	88.29	89.96	89.74	90.24	83.49	83.14	83.83	95.20	95.37	95.05
**γ**	91.33	90.72	91.93	92.63	92.29	92.95	85.98	86.50	85.48	96.43	96.58	96.28

**FIGURE 5:**
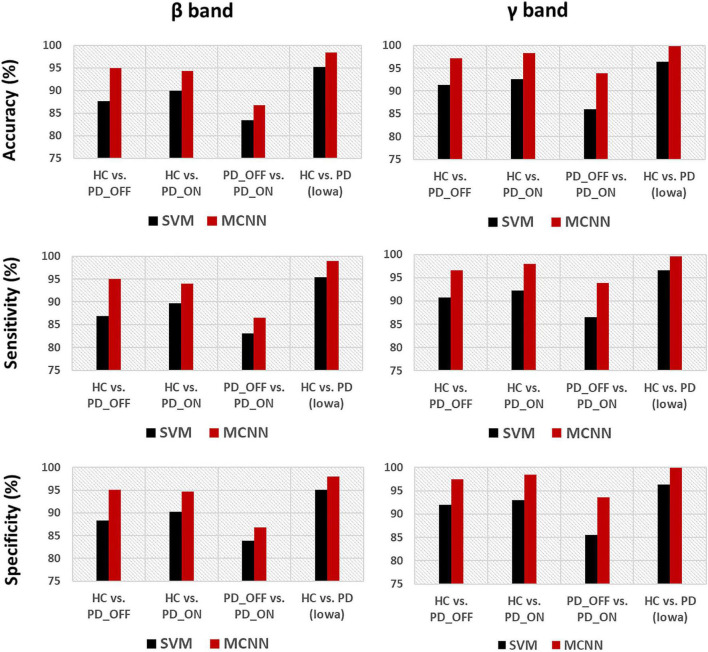
Accuracy, sensitivity, and specificity for the classification based on PSD+PLV features in the β and γ bands by using SVM and MCNN for the four comparison groups (i.e., HC vs. PD_OFF, HC vs. PD_ON, PD_OFF vs. PD_ON in UC San Diego dataset, and HC vs. PD in Iowa dataset).

In the third part of this study, we proposed a DL-based automatic PD detection model, the MCNN model, to classify PD (PD_OFF and PD_ON) and HC based on PSD+PLV features. Here we applied the MCNN model to perform two-class and three-class classifications on the UC San Diego dataset, respectively, and perform two-class classifications on the Iowa dataset. All results are ten-fold cross-validated, and the results for two-class classifications are shown in Table 6. It can be observed from Table 6, our proposed MCNN model can not only effectively discriminate PD (including PD_OFF and PD_ON) and HC, but also provides better classification performance than the SVM method. Similar to the SVM results, the best classification results appeared in the β and γ bands, especially the γ band, with over 90% accuracy, sensitivity, and specificity. The best classification performance was in HC vs. PD (Iowa dataset) with 99.75% accuracy, 99.42% sensitivity and 99.74% specificity. Figure 5 shows the comparison of the classification accuracy, sensitivity and specificity of SVM and MCNN based on PSD+PLV in β and γ bands. As can be seen from Figure 5, in the β and γ bands, the classification results of MCNN model outperformed SVM model in all cases. The case of PD_OFF vs. PD_ON (γ band) had the largest improvement, with 7.87%, 7.37%, and 8.16% higher accuracy, sensitivity and specificity than SVM.

**TABLE 6:** The results of two-class classifications based on PSD+PLV features in the two datasets by using MCNN model (unit: %, Accu. = Accuracy, Sens. = Sensitivity, Spec. = Specificity).

	HC vs. PD_OFF (UC San Diego dataset)			HC vs. PD_ON (UC San Diego dataset)			PD_OFF vs. PD_ON (UC San Diego dataset)			HC vs. PD (Iowa dataset)		
	Accu.	Sens.	Spec.	Accu.	Sens.	Spec.	Accu.	Sens.	Spec.	Accu.	Sens.	Spec.
**δ**	74.59	73.23	75.86	70.67	67.20	73.74	63.45	67.37	59.24	71.96	68.71	74.63
**θ**	80.63	79.52	81.57	78.45	78.09	78.61	65.99	71.92	59.93	84.91	83.24	86.74
**α**	82.73	80.10	85.18	79.96	76.67	82.81	69.03	74.93	62.72	92.39	91.72	92.93
**β**	95.01	95.03	95.04	94.40	93.98	94.77	86.78	86.55	86.87	98.47	98.94	98.03
**γ**	97.15	96.62	97.47	98.27	98.01	98.49	93.85	93.87	93.64	99.82	99.68	99.91

**FIGURE 6:**
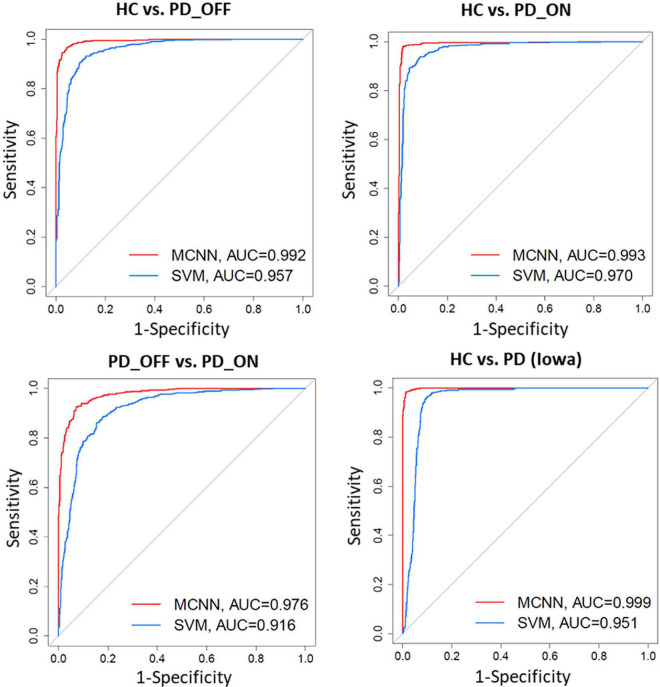
Receiver operating characteristic curve (ROC) and their Area Under Curves (AUC) for the SVM and proposed MCNN model used to classify subjects into HC, and PD (PD_OFF and PD_ON) based on PSD+PLV features.

In addition, we also compared the ROC curves and their corresponding AUC of the SVM and MCNN models in the two-class classification based on the PSD+PLV feature of the γ-band. As shown in Figure 6, In the four-group classification based on PSD+PLV features, the AUCs of ROCs obtained by SVM model are 0.957 (HC vs. PD_OFF), 0.970 (HC vs. PD_ON), 0.916 (PD_OFF vs. PD_ON), and 0.951 (HC vs. PD), respectively, while the AUCs of ROCs obtained by the proposed MCNN model are 0.992 (HC vs. PD_OFF), 0.993 (HC vs. PD_ON), 0.976 (PD_OFF vs. PD_ON), and 0.999 (HC vs. PD), respectively. Obviously, the proposed MCNN model can effectively utilize complementary multi-pattern features (PSD+PLV) to accurately detect PD patients (including PD_OFF and PD_ON), and perform better than the traditional SVM model.

**FIGURE 7:**
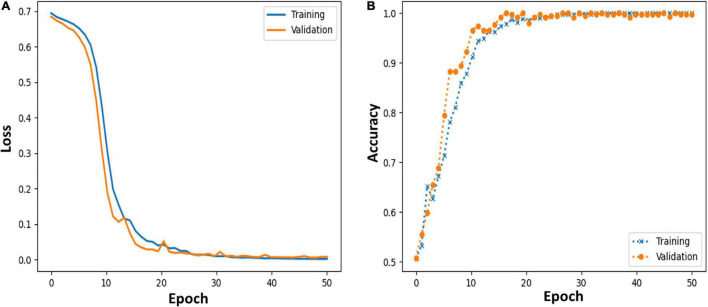
**(A,B)** Loss and accuracy of MCNN models during training and testing process in HC vs. PD (Iowa dataset) classification based on γ-band PSD+PLV features.

Moreover, we further applied the proposed MCNN to perform three-classification experiments on HC, PD_OFF and PD_ON in UC San Diego dataset based on the PSD+PLV hybrid feature, and the accuracy rates obtained in the five frequency bands were 68.71% (δ band), 79.20% (θ band), 82.42% (α band), 94.77% (β band), and 95.50% (γ band). In order to verify the reliability of the proposed MCNN model, Figure 7 shows the training and testing process of the PD vs. HC (Iowa dataset) classification experiment of the MCNN model based on the PSD+PLV features of the γ-band, where the ratio of the training set and the testing set was 9: 1. It can be observed that the MCNN model takes about 20 epochs to reach its maximum performance, i.e., the highest accuracy (Figure 7A) and the smallest loss (Figure 7B).

## Discussion

There is still a lack of consensus on the brain characterization of PD, and a more efficient and robust PD detection method is needed. Therefore, in this study, we performed a multi-pattern analysis based on brain activation and brain functional connectivity on resting-state EEG data of PD patients to explore abnormal brain activity in PD patients, and proposed a new PD detection model, MCNN model, to achieve high-precision identification of PD patients.

Findings from previous studies based on various features in the time, frequency, and time-frequency domains suggest that the resting-state EEG of PD patients was altered compared to HCs, and that both EEG-based spectral and connectivity markers are helpful in distinguishing HC and PD patients (Cozac et al., 2016). However, most of these studies are only based on the analysis of a single pattern (spectral or connectivity) to study the brain activity of PD and identify PD, which is difficult to fully reflect the brain functional characteristics of PD patients. In the present study, we performed a multi-pattern analysis of spontaneous brain activity in PD patients with PSD and PLV representing brain activation and brain functional connectivity, respectively. Among them, PSD can reflect the power distribution of spontaneous brain activity in each brain region, and PLV can reflect the information interaction between brain regions, both of which have been proved to be helpful for PD detection (Lee et al., 2019; Anjum et al., 2020). The results of our multi-pattern analysis showed that the HC group and PD group in the two datasets were significantly different in PSD (Figure 2) and PLV (Figure 3) features, especially in the β and γ bands. Specifically, PD patients had slightly weaker functional connectivity and frontal activation than HC in β and γ bands, while PD patients without medications exhibited stronger functional connectivity than PD without medications in most brain regions and local activation in β and γ bands. Our results are consistent with the findings of some previous studies. E.g., Gong et al. (2021) found abnormal phase-amplitude coupling between β and broadband-γ activities in PD patients based on resting EEG data from PD patients (Gong et al., 2021). Pezard et al. (2001) found that PD patients have reduced power in the β band by investigating non-linear properties of multichannel EEG in the early stages of PD. Wan et al. (2020) analyzed the PSD and phase lag index (PLI) of different sub-bands of the resting-state EEG in PD patients and found that β and γ rhythms in PD patients exhibited lower relative powers as compared to the HC group. There were also significant differences in the synchronization of the β bands between the two groups (Wan et al., 2020). George et al. (2013) found that dopaminergic therapy in PD decreases cortical β band coherence in the resting state. In addition, the classification results based on SVM also verified that PSD and PLV are very effective in the identification of PD patients (see Tables 3, 4). Among them, the classification accuracy based on PSD features is as high as 82.33%, and the classification accuracy based on PLV features is as high as 94.36%. It can also be observed from the results that the classification performance of PLV features is generally better than that of PSD features, which confirmed the conclusion of previous studies that phase features play a greater role than spectral power in the model classification (Lee et al., 2021). The above findings may imply that spectral and connectivity analysis of spontaneous EEG activity, especially in the β and γ bands, may be useful for the characterization and the accurate detection of PD.

The brain activation patterns capture activity differences among multiple brain regions, while the functional connectivity pattern reflect information interactions between brain regions. The activation distribution and connectivity patterns are complementary, reflecting different aspects of brain function. It has been demonstrated that the performance of emotion recognition can be improved by combining power spectral activation patterns and phase-related connectivity patterns based on EEG data (Li et al., 2019). Compared with single-pattern features, multi- pattern features are more likely to improve PD detection accuracy (i.e., provide more discriminative information). However, few studies have combined these two complementary patterns for PD detection. In this study, we combined PSD features and PLV features to distinguish HC from PD patients, and found that compared with PSD and PLV alone, based on hybrid features (i.e., PSD+PLV) can effectively improve the performance of PD detection (see Figure 4 and Table 5). Among them, HC vs. PD in the Iowa dataset obtained a high classification performance dataset with 96.43% accuracy, 96.58% sensitivity, 96.28% specificity and AUC 0.951 using the SVM model based on PSD+PLV features (γ band). Our results imply that integrating information from local power activities and network patterns helps improve the performance of PD detection.

There is much evidence that EEG data and ML or DL techniques can accurately identify disease characteristics or risk, which is promising for patients with brain disorders such as PD. However, there is still a lack of effective and robust automatic PD detection models. To this end, we proposed a novel deep learning model, multi-scale CNN, to achieve high-performance detection of PD patients. The MCNN model was designed based on the classic CNN model LeNet-5 (Lecun et al., 1998), on which the idea of multi-scale convolution and residual learning was added. The proposed MCNN can not only simultaneously extract multi-scale discriminative information in input features, but also improve the network degradation problem caused by deeper layers in deep learning. The experimental results (Figures 5−7 and Table 6) show that the proposed MCNN model can effectively identify PD (including PD_OFF and PD_ON) in both datasets, and has higher classification performance than the SVM method. The classification accuracy, sensitivity, specificity and AUC of the MCNN model for PD (including PD_OFF and PD_ON) and HC, as well as PD_OFF and PD_ON, based on PSD+PLV features (γ band) in both datasets are all over 93%. Among them, HC vs. PD (Iowa dataset) achieved the highest classification performance of over 99% in accuracy, sensitivity, specificity and AUC. Moreover, in the three-class classifications on HC, PD_OFF and PD_ON, MCNN obtained a high accuracy of 95.50%. The results based on the MCNN model are comparable to other state-of-the-art techniques developed for automated PD detection using the same EEG database, as shown in Table 7. Moreover, we also compared the performance of the traditional LeNet-5 network and the proposed MCNN model on PD detection, and found that the proposed MCNN model showed better classification results than the traditional LeNet-5 network in both datasets (including accuracy, sensitivity, and specificity), which proves that our improvements based on the LeNet-5 network are beneficial for PD detection.

**TABLE 7:** Summary of comparison of our work with other state-of-art techniques developed for automated Parkinson’s disease (PD) detection using the same electroencephalography (EEG) database.

Work	Dataset	Feature analysis	Machine and deep learning techniques	Classification	Accuracy
Khare et al., 2021a	UC San Diego	Tunable Q wavelet transform	Least square SVM	HC vs. PD_OFF	96.13%
				HC vs. PD_ON	97.65%
Khare et al., 2021b	UC San Diego	Wigner-ville distribution based spectrogram generation	2D CNN	HC vs. PD_OFF	99.7%
				HC vs. PD_ON	100%
Loh et al., 2021	UC San Diego	Gabor transform	2D CNN	HC vs. PD_OFF	99.44%
				HC vs. PD_ON	92.60%
				HC vs. PD_OFF vs. PD_ON	99.46%
Barua et al., 2021	UC San Diego	Aspirin pattern, statistical moments, and maximum absolute pooling	k nearest neighbor	HC vs. PD_OFF	99.93%
				HC vs. PD_ON	100%
Shaban, 2021	UC San Diego	three spatial channels	Artificial Neural Networks	HC vs. PD_OFF	98%
Shaban and Amara, 2022	UC San Diego	Continuous wavelet transforms	2D CNN	HC vs. PD_OFF	99.9%
				HC vs. PD_ON	99.8%
				HC vs. PD_OFF vs. PD_ON	99.6%
Anjum et al., 2020	Iowa	Linear predictive coding	Hyperplanes	HC vs. PD	85.7%
Lee et al., 2022	Iowa	Hjorth parameter and Gradient boosting decision tree	Gradient boosting decision tree	HC vs. PD	89.3%
Our work	UC San Diego	Phase locking value and Power spectral density	LeNet-5	HC vs. PD_OFF	89.16%
				HC vs. PD_ON	92.19%
				PD_OFF vs. PD_ON	86.56%
				HC vs. PD_OFF vs. PD_ON	84.38%
	Iowa			HC vs. PD	96.31%
Our work	UC San Diego	Phase locking value and Power spectral density	Proposed MCNN	HC vs. PD_OFF	97.15%
				HC vs. PD_ON	98.27%
				PD_OFF vs. PD_ON	93.85%

There are currently few studies using the Iowa dataset (14 PD patients and 14 controls), for which our proposed MCNN model has a large improvement in classification performance compared to other related studies (Anjum et al., 2020; Lee et al., 2022). E.g., our accuracy is about 14.12% higher than the linear-predictive-coding EEG algorithm proposed by Anjum et al. (2020), and about 10.52% higher than the Hjorth parameter and the gradient boosting decision tree algorithm proposed by Lee et al. (2022). For the UC San Diego dataset (15 PD patients and 16 controls), the classification performance (accuracy, sensitivity, specificity, and AUC are above 93% in γ band) of our proposed MCNN model is comparable, although not the highest, compared to other related studies (Khare et al., 2021a,b; Loh et al., 2021; Shaban, 2021; Shaban and Amara, 2022). Some previous work can achieve close to 100% accuracy (Barua et al., 2021; Khare et al., 2021b). E.g., Khare et al. (2021b) obtained high accuracies of 99.9% and 100% on HC vs. PD_OFF and HC vs. PD_ON using 2D-CNN based on the smoothed pseudo-Wigner Ville distribution, respectively. Barua et al. (2021) achieved 99.93% and 100% classification accuracy for HC vs. PD_OFF and HC vs. PD_ON using the proposed novel aspirin pattern, respectively. Shaban and Amara (2022) used a continuous wavelet-based deep learning method to obtain a high accuracy of 99.9% for PD detection. Although these studies achieved slightly higher accuracy than our proposed model, their methods were only validated on a single dataset and did not generalize to other datasets. The good performance of our proposed MCNN model for PD detection was verified in two completely independent datasets (i.e., UC San Diego dataset and Iowa dataset) and many different classification experiments (including HC vs. PD_OFF, HC vs. PD_ON and PD_OFF vs. PD_ON).

Our study showed promising results in PD detection, however, the following limitations still exist. First, the sample size of this study is small. Although we have analyzed two datasets (29 PD patients in total, 30 HCs), the sample size is small for a deep learning study. Second, we integrated information from local power activities and network patterns by splicing features, ignoring the fact that information from these two patterns may be redundant. Finally, our proposed PD detection model lacks real-world clinical and experimental validation. For these limitations, in future studies, we will strive to advance collaboration with hospitals, hoping to collect more clinical data and validate our proposed PD detection model in real-time patient-generated data. Furthermore, we will explore efficient feature fusion methods to fully utilize the information of activation patterns and connection patterns to further improve PD detection performance.

## Conclusion

In this study, a multi-pattern analysis based on brain activation and brain functional connectivity was performed on the brain functional activity of PD patients, and a novel PD detection model based on multi-scale CNN was proposed. Based on the results in two independent resting-state EEG datasets, PSD and PLV features in the β and γ bands may characterize PD and be effective for PD (including OFF and ON medications) detection. Moreover, the combined use of brain activation and functional connectivity, two patterns with compensatory information, can effectively improve the performance of PD detection. Furthermore, our proposed multi-scale CNN model shows great potential for automatic PD detection with high cross-validation accuracy and AUC of 99% for sensitivity, specificity and ROC. Our study may help to further understand the characteristics of PD and provide new ideas for future PD diagnosis based on spontaneous EEG activity.

## Data availability statement

Publicly available datasets were analyzed in this study. The UC San Diego dataset analyzed for this study can be found in the following link: https://openneuro.org/datasets/ds002778/versions/1.0.4. The Iowa dataset analyzed for this study can be found in the following link: https://bit.ly/3pP1pts.

## Author contributions

LQ and JP designed the experiments and wrote the manuscript. JL collected and analyzed the data. All authors contributed to manuscript revision, read, and approved the submitted version.
